# Hydrocephalus and Cerebral Small Vessel Disease in an Adult With Meningococcal Meningitis and Dilated Cardiomyopathy: A Fatal Outcome

**DOI:** 10.1002/ccr3.70343

**Published:** 2025-03-24

**Authors:** Yousif Mohamed, Ahmed Mohamed, Abubaker Elemam, Abubaker Babiker

**Affiliations:** ^1^ Department of Radiology University of Khartoum Khartoum Sudan; ^2^ Department of Internal Medicine University of Khartoum Khartoum Sudan; ^3^ Department of Interventional Radiology Washington University Washington District of Columbia USA; ^4^ Department of Radiology University of Al Qadarif Al Qadarif Sudan

**Keywords:** cerebral small vessel disease, dilated cardiomyopathy, hydrocephalus, meningococcal meningitis

## Abstract

Meningococcal meningitis can lead to serious neurological complications such as hydrocephalus directly and contribute to cerebral small vessel disease indirectly, especially in patients with pre‐existing conditions like dilated cardiomyopathy. Timely identification and aggressive treatment are essential, although the prognosis is often poor when numerous complications are present.

## 1 Introduction

Bacterial meningitis pathogens shift with age—neonates face *Escherichia coli* and Group B *Streptococcus*, while older individuals are more affected by *Streptococcus pneumoniae*, *Neisseria meningitidis* , and *Haemophilus influenzae* [1]. Meningococcal meningitis is a kind of bacterial meningitis caused by the bacterium *Neisseria meningitidis* . It is a medical emergency where symptoms may range from transient fever to fulminant bacteremia and septic shock [2].

Among the 12 serogroups, A, B, C, W, X, and Y are the primary serogroups associated with meningococcal disease, with approximately 1.2 million cases occurring worldwide each year, resulting in 135,000 fatalities and a mortality rate of 11%, contributing to notable illness and death [3]. Since its introduction in 2010, MenAfriVac has been highly effective in reducing the cases and carriage of serogroup A—the previously predominant serogroup responsible for the majority of meningococcal diseases within the region—by over 99%. This in turn has resulted in a great reduction of outbreaks caused by this serogroup, especially in areas that have been vaccinated [4]. Since then, a number of cases have increased that were caused by other serogroups. In 2022, an epidemic due to a new strain of serogroup C was reported in Niger, which indicated that the epidemiological landscape had shifted [5].

Hydrocephalus occurs in 5% of adults with community‐acquired bacterial meningitis and is linked to elevated mortality rates [6].

Cerebral small vessel disease (CSVD) is a broad term that encompasses intracranial vascular disorders arising from various pathological and neurological mechanisms, along with a syndrome that describes multiple clinical symptoms and neuroimaging characteristics resulting from structural changes in both vascular and brain tissue. The onset of cerebrovascular changes represents a significant intracranial complication during acute bacterial meningitis and is linked to unfavorable outcomes [7].

The strongest risk factors for an unfavorable outcome are those that are indicative of systemic compromise [8]. Patients with dilated cardiomyopathy (DCM) have compromised cardiac function and reduced cerebral perfusion, increasing the risk of ischemic injury during episodes of hypotension or shock. This can lead to long‐term cognitive deficits or other neurological impairments [9].

The uniqueness lies in the combination of hydrocephalus, CSVD, and DCM in relation to meningococcal meningitis; although each one of those conditions has a defined pathophysiology, their coexistence in one patient raises the issue of appropriateness regarding evaluation and treatment. It would not only contribute to furthering the outcome but also to the individualization of treatment.

## 2 Case History and Examination

A 51‐year‐old male hypertensive with a 5‐year history of DCM and an unavailable vaccination record was rushed to Al Karama Hospital, Sinnar, with a new onset decrease in level of consciousness. His co‐patients described him as having a syncopal event and falling on his head 1 day before admission. They also mentioned that the patient complained of severe headache, dizziness, fever, vomiting, and slurred speech.

Upon arrival, the patient appeared ill and febrile (39.7°C). Tachycardic and hypotensive (100/70 mmHg). The patient exhibited alternating periods of hyperventilation and apnea. His Glasgow Coma Scale was 6/15. The neurological assessment revealed paralysis and hypertonia in both lower extremities, sluggish pupillary reactions, and nuchal rigidity, with positive Babinski and Kernig signs consistent with meningitis and suspicion of raised intracranial pressure. Signs of DCM were present: “bilateral lower limb edema, displaced apical impulse, and third heart sound gallop.” These findings were consistent with decompensated heart failure secondary to DCM.

## 3 Differential Diagnosis, Investigations, and Treatment

Laboratory investigations (Table 1) were within normal limits except for leukocytosis of 15,000 and mostly neutrophils (80%) and elevated C‐reactive protein level (CRP) of 25 mg/dL.

**TABLE 1:** Showing laboratory investigations of the patient.

Parameter	Patient value	Reference range
Total WBCs	15.2 × 10^3^/μL	3.5–11 × 10^3^/μL
Neutrophils %	86%	50%–70%
Lymphocytes %	09%	20%–40%
Total RBCs	4.42 × 10^6^/μL	4.5–5.9 × 10^6^/μL
Hemoglobin	12.8 g/dL	13–17 g/dL
Platelet count	405 × 10^3^/μL	150–450 × 10^3^/μL
Blood urea	52 mg/dL	10–50 mg/dL
Serum creatinine	1.2 mg/dL	07–1.4 mg/dL
Serum sodium	142 mmol/L	135–145 mmol/L
Serum potassium	4.06 mmol/L	3.5–5.0 mmol/L
CRP	25 mg/dL	< 6 mg/dL
Urine cast	Granular and hyaline	Nil
CSF total WBCs	100 c/mm^3^	< 5 c/mm^3^
Neutrophils %	50%	
Lymphocyte %	32%	
CSF sugar	35 mg/dL	50–80 mg/dL
CSF protein	5.3 g/dL	< 4.5 g/dL
LV Ejection fracure on echocardiogram	10%	50%–70%

**FIGURE 1:**
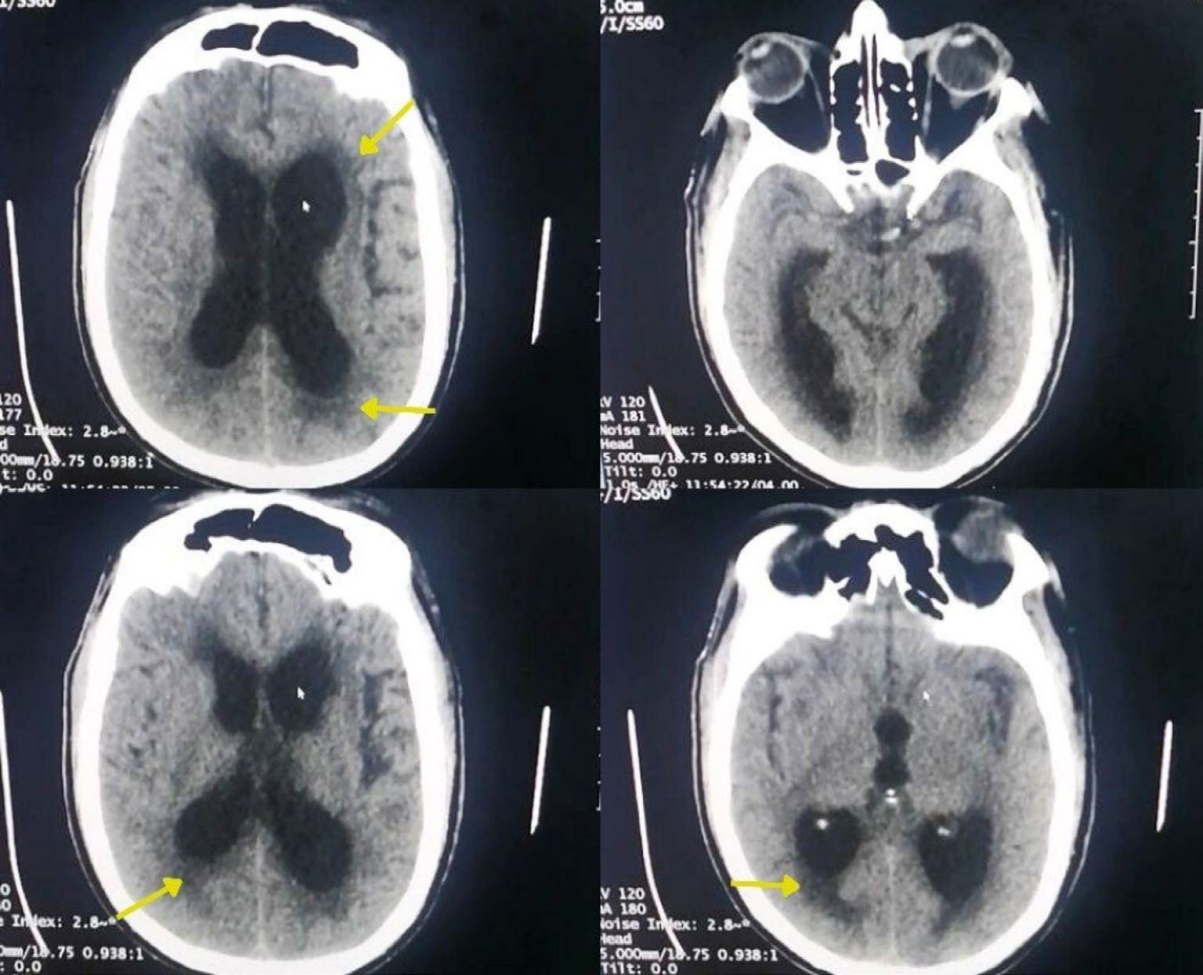
Axial CT without contrast reveals enlarged lateral and third ventricles, along with the effacement of sulci and widening of the Sylvian fissure. Additionally, periventricular hypodensities are noted (indicated by yellow arrows).

A non‐contrast axial computed tomography (CT) of the brain showed enlarged lateral and third ventricles with effacement of sulci. Bleeding in Sylvian cisterns and midline shift were not observed. Also, periventricular hypodensity was observed; this was correlated with the patient's history with vascular risk factors and diagnosed as CSVD (Figure 1), but due to the limited access of MRI and the clinical urgency of the case, this was not further confirmed.

A nasogastric tube was placed for feeding, and his previous chronic medications (amlodipine 5 mg daily, losartan 25 mg daily, carvedilol 12.5 mg twice daily, furosemide 40 mg daily, and spironolactone 50 mg daily). IV broad‐spectrum antibiotics (ceftriaxone and vancomycin), dexamethasone, and mannitol were given. A spinal tap was performed to analyze the cerebrospinal fluid (CSF), identify the causative organism, and provide targeted therapy. The procedure was performed by an experienced physician in a controlled setting with the immediate availability of resuscitation equipment, and 8 cc of turbid CSF was withdrawn, and increased opening pressure was observed, after which a change in respiratory pattern was noted. CSF gram staining revealed gram‐negative diplococci and other features suggestive of meningococcal meningitis. An external ventricular drain was placed, and 300 mL of CSF was removed as the patient wasn't fit for a ventriculo‐peritoneal shunt.

## 4 Outcome

Unfortunately, the patient's condition deteriorated despite aggressive medical and neurosurgical interventions. He suffered cardiac arrest and passed away 1 day after the external ventricular drain placement.

## 5 Discussion

This case illustrates the severe complications of meningococcal meningitis in the setting of pre‐existing cardiovascular disease. Hydrocephalus and CSVD are well‐known neurological sequelae of bacterial meningitis.

Meningococcal meningitis remains among the serious public health concerns throughout the world, especially in the areas within the meningitis belt, like Sudan [10]. While vaccination has gone a long way in considerably reducing the burden of serogroup A infections, challenges related to sufficiently high coverage, coupled with environmental factors, remain to be addressed appropriately in order to prevent outbreaks in the future [11]. Management of this important public health problem involves continued surveillance along with public health responses.

The occurrence of CSVD‐like imaging findings in patients with meningitis is uncommon. When present, these findings are typically associated with prolonged inflammation or microvascular compromise resulting from the infection. Other cerebrovascular complications are frequently found in patients with bacterial meningitis [12] and consist of thrombosis, vasculitis, acute cerebral hemorrhage, and mycotic aneurysm formation of large, medium, or small cerebral vessels [13]. Although this was not confirmed in this report due to limited diagnostic resources, it was reported to occur in approximately one‐fifth of patients [14] and is associated with worse outcomes.

On the other hand, hydrocephalus complicates community‐acquired bacterial meningitis, with most cases diagnosed as communicating hydrocephalus due to impaired CSF absorption at the arachnoid granulation [6]. Hydrocephalus is associated with high fatality rates; in one study, 50% of patients experienced mortality and poor outcomes despite receiving neurosurgical intervention like CSF shunting [6]. Distrurbed consciousness and old age at the time of admission were reported to be associated with poor disease outcome [15].

Although the exact relationship between meningococcal meningitis and DCM has not been established, several mechanisms suggest possible links.

Meningococcal sepsis can lead to significant myocardial depression. This is due to the release of inflammatory cytokines like interleukin‐6, which has been reported to predict the extent of myocardial dysfunction in children with meningococcal disease [16]. High levels of such cytokines may lead to increased vascular permeability and endothelial cell dysfunction; this may further exacerbate pre‐existing DCM [17].

Most studies indicate that cardiac outputs in septic patients, as with meningococcal infection, are generally low and filling pressures high, compared to cardiac outputs in other forms of sepsis [18]. Furthermore, inadequate cardiac function may worsen the hemodynamic instability associated with severe infections like bacterial meningitis. Research has shown that individuals with existing heart issues, such as DCM, could face higher mortality rates due to the compounded effects of septic shock combined with cardiac impairment [19].

This report serves as a grave reminder of the devastating consequences of meningococcal meningitis, particularly in individuals with pre‐existing cardiovascular conditions. Meningococcal meningitis continues to pose a public health challenge, especially in areas like Sudan, where vaccination coverage is likely inadequate, highlighting the necessity for vaccination and early interventions to avert serious complications. The report is beneficial for managing complex cases with involvement of multiple organ systems. It emphasizes the need for tailored treatment strategies and the difficulties faced in caring for patients with limited access to advanced diagnostic tools (e.g., MRI). Finally, this report enhances the understanding of the complex relationship between systemic and neurological complications arising from severe infections.

## Author Contributions

**Yousif Mohamed:** conceptualization, data curation, formal analysis, investigation, methodology, project administration, resources, supervision, validation, writing – original draft, writing – review and editing. **Ahmed Mohamed:** resources, supervision, validation, writing – original draft, writing – review and editing. **Abubaker Elemam:** supervision, writing – review and editing. **Abubaker Babiker:** visualization, writing – review and editing.

## Consent

Written informed consent was obtained from the patient's relatives for publication of this case report and accompanying images.

## Data Availability

The authors have nothing to report.
